# Bridging the Gap: A Novel Application of Corneal Collagen Cross-Linking in Treating Recurrent Corneal Erosions Secondary to Wound Gaping After Astigmatic Keratotomy

**DOI:** 10.7759/cureus.11018

**Published:** 2020-10-18

**Authors:** Christopher M Anthony, Adam Altman, Timothy Soeken, Gary L Legault

**Affiliations:** 1 Ophthalmology, San Antonio Uniformed Services Health Education Consortium, San Antonio, USA; 2 Ophthalmology, Wilford Hall Ambulatory Surgical Center, San Antonio, USA; 3 Ophthalmology, Brooke Army Medical Center, San Antonio, USA

**Keywords:** crosslinking, corneal erosions, astigmatic keratotomy

## Abstract

Corneal collagen cross-linking is a minimally invasive therapeutic technique indicated for the treatment of keratoectasia. Recently, it has also been utilized for a variety of other ophthalmologic conditions ranging from infectious keratitis to corneal edema. We report the novel application of corneal collagen cross-linking in the treatment of recurrent corneal erosions secondary to wound gaping after astigmatic keratotomy (AK).

## Introduction

Corneal collagen cross-linking is a therapeutic technique approved in 2016 by the United States Food and Drug Administration for halting the progression of keratoconus and post-LASIK ectasia. As a minimally invasive technique, it utilizes riboflavin drops activated by ultraviolet light. Once activated, the riboflavin forms reactive oxygen species and additional covalent bonds between corneal collagen molecules. The additional covalent bonds formed between collagen molecules result in biomechanical stiffening and, thus, increased stability of the cornea.

Although originally designed to treat keratoectasia, collagen cross-linking has also been utilized in recent years for the treatment of a variety of ophthalmologic conditions including refractive surgery-induced keratoectasia, infectious keratitis, corneal edema, and pellucid marginal degeneration [1-2]. We report the novel application of corneal collagen cross-linking in the treatment of recurrent corneal erosions secondary to wound gaping after astigmatic keratotomy.

## Case presentation

A 57-year-old female with past ocular history of an eight-cut radial keratotomy (RK) with two transverse incision astigmatic keratotomy (AK) in both eyes and subsequent recurrent corneal erosions (RCE) presented to our ophthalmology clinic complaining of sharp pain and photophobia in the left eye. Notably, she experienced five episodes within the last 12 months of RCEs secondary to the temporal AK of the left eye (OS). Prior treatment for RCEs included bandage contact lenses (BCLs), moxifloxacin, sodium chloride hypertonicity solution, artificial tears, cyclosporine, and most recently anterior stromal puncture. 

Screening was within normal limits, with the exception of best-corrected visual acuity (BCVA) being diminished during acute events but returning to 20/20 between episodes. On slit-lamp examination, the patient was noted to have a recurrent corneal erosion with underlying gaping AK inferotemporally of the left cornea. Dilated fundoscopic examination was unremarkable. Initial therapy included BCL, moxifloxacin, sodium chloride hypertonicity solution, and continuation of cyclosporine.

**Figure 1:**
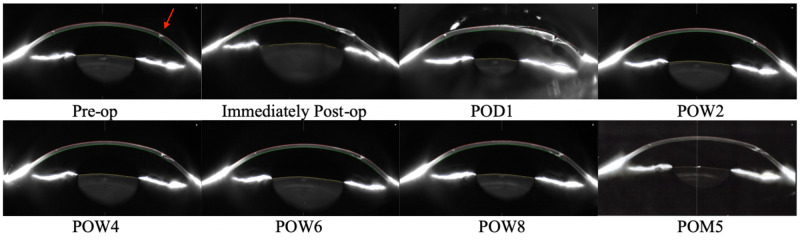
Pre-operative and post-operative Scheimpflug images Upper-left to lower-right: Images 1-8. Image 1 is a pre-operative Scheimpflug image (SI) displaying asymmetric enlargement of temporal astigmatic keratotomy (AK) in the left eye (OS). Image 2 is an immediately post-operative SI displaying temporal AK wound apposition, mild corneal edema, and a well-formed Anterior Chamber (AC) OS. Image 3 is a post-op day (POD) #1 SI displaying gaping temporal AK wound with overlying ReSure, corneal edema, and a well-formed AC OS. Image 4 is a post-op week (POW) #2 SI displaying gaping temporal AK wound with absence of ReSure, resolved corneal edema, and closed epithelium OS. Image 5 is a POW#4 SI displaying narrowing of gaping temporal AK wound and stability of closed epithelium OS. Images 6 – 8 are SI displaying stability of temporal AK wound and epithelium OS at POW#6, POW#8, and post-op month (POM) #5, respectively.

During follow-up over the next several months, the patient reported fluctuating symptoms, denied any subjective improvement, and continued to demonstrate unresolved edema surrounding the temporal AK OS despite management with antibiotics, steroids, topical cyclosporine, artificial tears, sodium chloride ointment, BCLs, and even anterior stromal puncture. Significantly, underlying the area of erosion was a gaping AK incision as seen in image one of Figure 1.

Due to the persistent nature of the patient’s subjective complaints and increasing frequency of recurrent corneal erosions secondary to gaping AK incision, the patient was counseled and offered a re-approximation of the AK incision using 11-0 suture followed by collagen cross-linking in order to reduce further surgical trauma and promote corneal stromal healing. The epithelium was debrided over the AK incision with alcohol and an 11-0 suture was placed through the AK incision to appose wound edges. Shortly after suture placement, trace aqueous humor was noted leaking from the suture track, however the globe was noted to be stable. Cross-linking was carried out with riboflavin and ultraviolet (UV) light application for 30 minutes in accordance with FDA approved collagen cross-linking Dresden protocol. Repeat images were obtained immediately following the procedure as seen in image two of Figure 1.

Initial post-operative exam again demonstrated trace fluid leaking around the suture, which was then removed, with subsequent application of ReSure (Ocular Therapeutix, Bedford, MA) and a BCL. Routine post-operative drops prescribed included antibiotics, artificial tears, topical steroid, and promethazine with oral vitamin C 500 mg twice a day.

On post-operative day (POD) #1, visual acuity OS was 20/400 (pinhole to 20/60-2) and fluorescein exam demonstrated wounds were Seidel negative. Trace eyelid edema, conjunctival injection, corneal edema, and anterior chamber reaction were present. POD#1 images reveal a gaping AK wound with ReSure in place, as seen in image three of Figure 1.

The remainder of her post-op course was notable for repeat ReSure application POD#4. Complete epithelium closure was noted at post-operative week (POW) #2 and remained closed throughout follow-up. Images four through seven of Figure 1 demonstrate continued resolution of corneal edema, epithelialization of the wound, and narrowing over the gaping AK.

Upon final examination at post-operative month (POM) #5, complete AK incision closure with intact overlying epithelium was noted as shown in image eight of Figure 1. The patient is currently only on artificial tears with uncorrected visual acuity (UCVA) 20/40-1 and BCVA 20/20+2 and remains free from RCE. 

## Discussion

The presenting symptoms of recurrent corneal erosions secondary to gaping keratotomy wounds are the same as for other etiologies of RCE in addition to abrasions and ulcers; notably pain, photophobia, and decreased vision. RCE etiologies include ocular trauma, underlying basement membrane dystrophy, stromal degeneration, or prior ocular surgery. The etiology of RCEs in our case was found with Pentacam and Anterior Segment optical coherence tomography (OCT), as displayed above, revealing a gaping keratotomy wound. Gaping wounds after keratotomy may be caused by patient factors including underlying collagen vascular disorder, post-op ocular massage, or movement during the procedure as well as surgical factors including perforation, inexperience, repeat enhancements, or crossing incisions [3]. 

One recent study estimates that ocular injury accounts for 45% - 64% of reported cases of recurrent corneal erosions [4]. The strong correlation between ocular insult and RCEs may be explained, at least in part, by the fact that corneal injury leads to inflammation with subsequent disruption of the epithelial basement membrane and weakening of the extracellular adhesion network. 

Current treatment options for recurrent corneal erosions are directed primarily towards decreasing inflammation and strengthening the extracellular adhesion network within the affected cornea. Conservative management consists of artificial tears, oral non-steroidal anti-inflammatories, cycloplegics, antibiotics, preservative-free lubricating drops, and/or hypertonic saline ointment. Further management for refractory cases includes matrix metalloproteinase inhibitors, topical corticosteroids, a bandage contact lens, and/or amniotic membrane graft. Surgical options are chosen based on the location of the erosion(s) relative to the visual axis and include anterior stromal puncture, alcohol delamination, epithelial debridement, diamond burr polishing, and/or phototherapeutic keratectomy (PTK) [4].

Our case demonstrates that collagen cross-linking may be a useful modality in treating refractory recurrent corneal erosions secondary to wound gaping after astigmatic keratotomy in addition to the armamentarium of conservative and surgical options currently available. Collagen cross-linking has been shown to strengthen collagen bonds within the cornea and improve corneal integrity and stability. A recent ex vivo study on cadaveric eyes demonstrated that the addition of cross-linking to an “ex vivo model of either penetrating or anterior lamellar keratoplasty led to an increase in adhesion strength of the donor-recipient corneal interface, evaluated using burst IOP and tissue separation force measurements" [5]. Furthermore, there is an abundance of evidence showing that cross-linking is an effective method of biomechanical corneal stabilization in RK-operative patients [6-7]. We propose that collagen cross-linking strengthened the existing extracellular collagen adhesion network within our patient’s cornea without repairing previously incised collagen fibers from prior AK and RK procedures, allowing for structural reinforcement of the AK incision and, consequently, resolution of the recurrent corneal erosions. 

Numerous factors may have contributed to the resolution of the RCE and decreased keratotomy width. Primarily, we propose that the collagen cross-linking allowed for additional collagen bonds to be formed and therefore strengthening of the wound. Additional factors include the keratectomy performed as part of the CXL, corneal edema which may have assisted in wound healing and approximation, brief placement and removal of an 11-0 suture, and two applications of ReSure over the wound. 

While this case addresses the potential use of collagen cross-linking in the treatment of RCE secondary to gaping AK, further studies designed to minimize the confounding factors listed above must be performed to elucidate the role of corneal collagen cross-linking in the treatment of RCEs. Additionally, we recommend collagen cross-linking be explored as a treatment modality for recurrent corneal erosions of other etiologies to include partial and full-thickness lacerations and other circumstances involving corneal gaping.

## Conclusions

Corneal collagen cross-linking is a versatile therapeutic technique used to treat an expanding variety of ophthalmologic conditions ranging from keratoectasia to infectious keratitis. Our case demonstrates that it may also have the potential to treat recurrent corneal erosion secondary to corneal insult such as wound gaping after astigmatic keratotomy. However, corneal collagen cross-linking should be explored more extensively as a treatment option for recurrent corneal erosion before it can be adopted into widespread use. We suggest that future applications of collagen cross-linking, such as corneal lacerations and other defects, be explored.
